# Volitional control of vocalizations in corvid songbirds

**DOI:** 10.1371/journal.pbio.3000375

**Published:** 2019-08-27

**Authors:** Katharina F. Brecht, Steffen R. Hage, Natalja Gavrilov, Andreas Nieder

**Affiliations:** 1 Animal Physiology Unit, Institute of Neurobiology, University of Tübingen, Tübingen, Germany; 2 Neurobiology of Vocal Communication, Werner Reichardt Centre for Integrative Neuroscience, University of Tübingen, Tübingen, Germany; University of Zürich, SWITZERLAND

## Abstract

Songbirds are renowned for their acoustically elaborate songs. However, it is unclear whether songbirds can cognitively control their vocal output. Here, we show that crows, songbirds of the corvid family, can be trained to exert control over their vocalizations. In a detection task, three male carrion crows rapidly learned to emit vocalizations in response to a visual cue with no inherent meaning (go trials) and to withhold vocalizations in response to another cue (catch trials). Two of these crows were then trained on a go/nogo task, with the cue colors reversed, in addition to being rewarded for withholding vocalizations to yet another cue (nogo trials). Vocalizations in response to the detection of the go cue were temporally precise and highly reliable in all three crows. Crows also quickly learned to withhold vocal output in nogo trials, showing that vocalizations were not produced by an anticipation of a food reward in correct trials. The results demonstrate that corvids can volitionally control the release and onset of their vocalizations, suggesting that songbird vocalizations are under cognitive control and can be decoupled from affective states.

## Abstract 2

Songbirds are renowned for their acoustically elaborate songs, but it is unclear whether they have cognitive control over their vocal output. Using operant conditioning, this study shows that carrion crows, songbirds of the corvid family, can exert control over their vocalizations.

## Introduction

Songbird vocalizations are elaborate and complex communicative signals whose behavioral and neuronal foundations have been extensively studied [1–3]. Similar to other birds’ vocalizations, they not only play an important role in reproduction and territory defense but also serve to ensure social cohesion, coordinate mobbing of predators or food recruitment, and allow individual recognition [4]. In contrast to the communicative signals of most animal taxa, songbirds’ vocalizations are learned by imitation [5–7] and show a degree of flexibility [8] in onset [9], social context [10,11], and structure [12,13]. This flexibility potentially indicates that songbird vocalizations are under volitional control. However, the observed context-dependent variability in avian vocalizations might simply be driven by involuntary mechanisms and need not be based on cognitive control. Indeed, changes in mobbing calls depending on the size of the mobbed predator [12] or food quality [14] can be explained by changes in arousal [15]. Here, we present a direct test of the conjecture that songbirds might volitionally control their vocalization in the sense that they can be emitted or inhibited at will, as opposed to being involuntary responses to food, mates, or predators and being largely dependent on affective states.

In order to demonstrate “volitional vocalizations,” three criteria have to be fulfilled in unison: First, vocalizations need to be uttered in response to an arbitrary instruction stimulus that is neutral in its value or emotional valence. Second, vocalizations need to be uttered in a manner that is temporally contingent to the instruction stimulus. Third, vocalizations need to be produced reliably after the presentation of the instructive stimulus and withheld in its absence or after the presentation of another instructive stimulus. This list of criteria is similarly applied in neuropsychological tests to differentiate between volitional and affective (emotional) responses in patients [16,17]. For example, patients with facial paralysis due to damage of descending pathways from the motor cortex have considerable difficulty smiling or frowning on command, a condition called “voluntary facial paresis,” even though they smile or frown spontaneously in response to their emotional state. Similar dissociations, in which some patients with neurological injuries may lose volitional control of their speech but can still laugh, scream, or groan when they are happy, frightened, or in pain, have been observed for vocalizations.

In determining whether songbirds can volitionally vocalize, we here adopt a paradigm that fulfills these criteria in order to distinguish volitional from affective vocalizations. Corvids are particularly well suited for such investigations because they are known for their sophisticated behavioral flexibility [18,19]. Importantly, as songbirds, corvids possess a large and flexible vocal repertoire [20,21] that transmits a range of information, such as an individual’s sex, age, and dominance [22]; the present behavioral context [23]; or third-party relationships [24]. Moreover, large-billed crows (*Corvus macrorhynchos*) [25] as well as house crows (*C*. *splendens*) [26] have the typical set of song nuclei characteristic for oscines.

## Results

### High performance in the detection task

**Fig 1:**
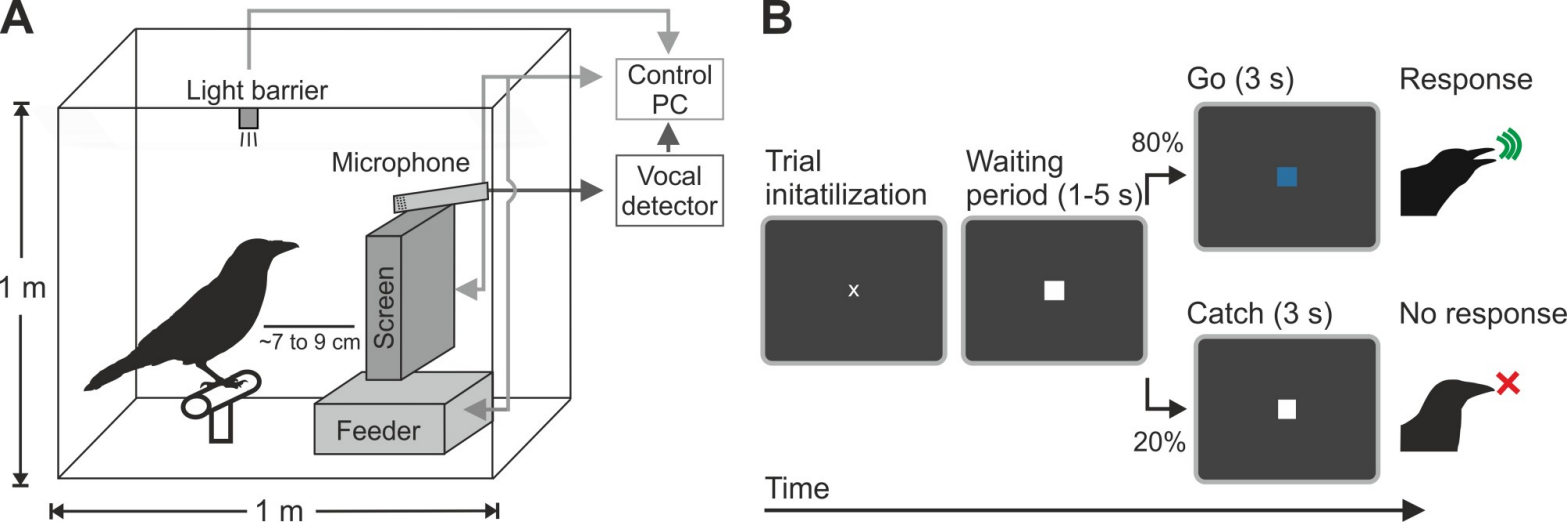
Schematic illustration of setup and task. (A) Carrion crows were trained in an operant chamber. They could initialize a trial by placing their head in a circumscribed position in front of a stimulation monitor (which was controlled by an infrared light barrier). The monitor presented the visual stimuli. Upon correct vocalization, crows were rewarded automatically by a feeder placed underneath the screen that presented the task. Vocalizations were recorded and analyzed online by a vocal detector. Stimulus presentation and behavioral control was accomplished by a control PC. (B) Visual detection task with vocalizations as response. Once the crows were positioned in front of the stimulation monitor, a white square appeared that indicated the variable waiting period. In 80% of the cases, a go cue (blue square; RGB values: 0, 0, 204) appeared that prompted the crow to vocalize in order to receive a reward. In the other 20% of the cases (catch trials), the white square remained after the waiting period had expired, and the crow was required to refrain from vocalizing. PC, personal computer; RGB, red–green–blue.

Three carrion crows were trained to vocalize in response to the presentation of a cue with no inherent meaning and to refrain from vocalizing when another cue was presented. In this first computerized detection task, the crows earned rewards by vocalizing in response to a specific visual cue (“go cue”). To initiate a trial, the crows had to position their head in front of the computer screen (**Fig 1A**). After a variable waiting period (1–5 seconds), a go cue prompted the crow to vocalize within the next 3 seconds (**Fig 1B**). Only vocalizations within 3 seconds after go cue onset were rewarded and counted as a “hit.” A vocalization in the waiting period led to the abortion of the trial and was followed by a time-out of 500 ms. Two of the crows were then retrained on a task in which they were confronted with the reversed color code of the previously learned task and with an additional nogo cue. Vocalizations were detected automatically by computer software and were recorded and stored to disc for offline analysis.

To ensure that vocalizations were indeed in response to the presentation of the go cue and not simply emitted after a certain waiting time had elapsed, 20% of trials were “catch” trials. In these catch trials, the waiting period, indicated by a white square, was not followed by a go cue. Instead, the waiting period continued, and the crows had to refrain from vocalizing for the duration of a go trial—that is, between 4 and 8 seconds (1–5 second waiting period plus 3 second “catch”). A vocalization in a catch trial was defined as a “false alarm” in the signal detection context and was followed by a time-out of 500 ms.

**Fig 2:**
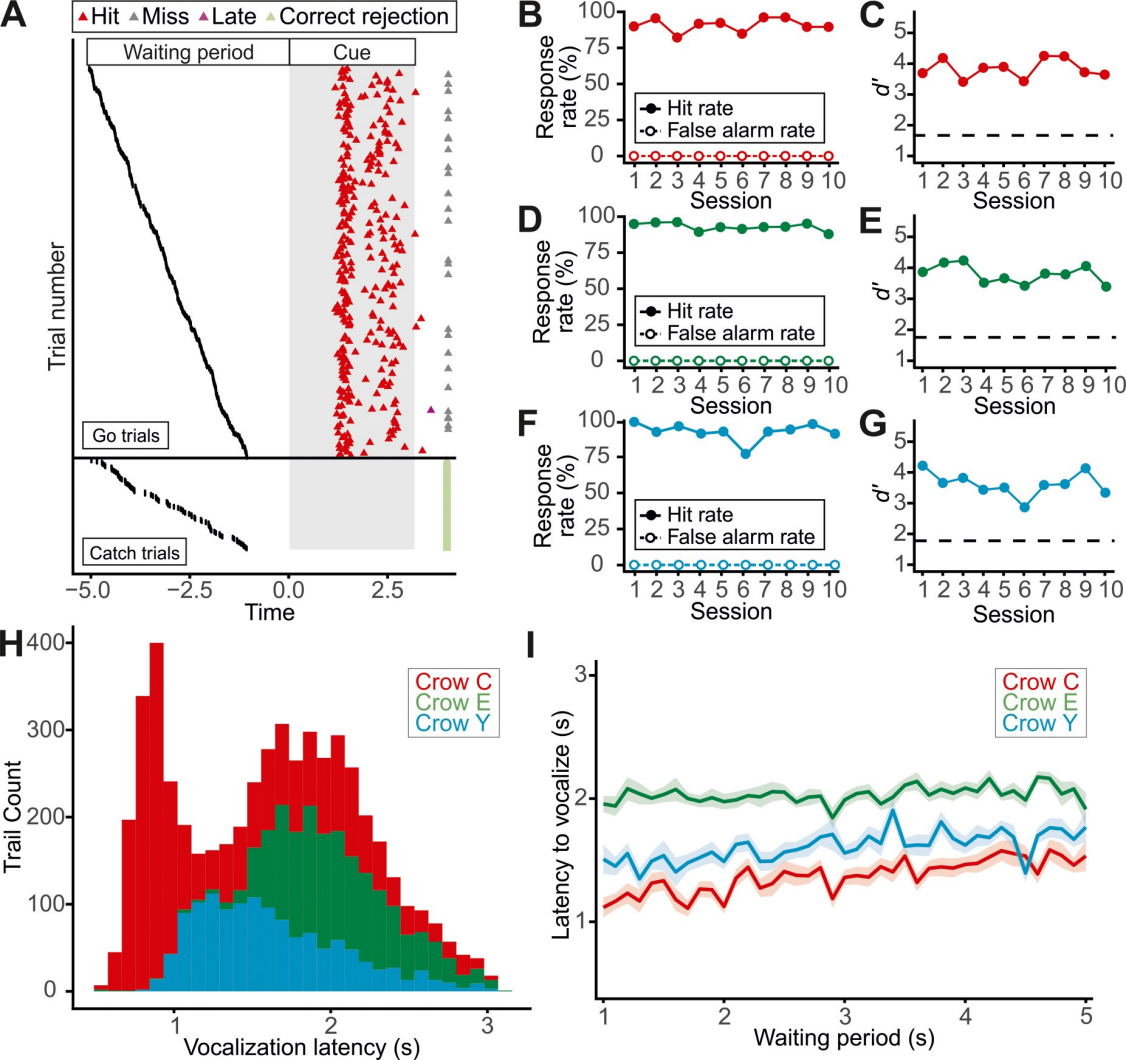
Task performance in the first detection task. (A) Detailed behavior of crow C in an exemplary session (session 4). The responses for all 300 trials, sorted according to length of the waiting period (black dots) for illustration, are shown. Each line represents one trial. Data are separated in catch and go trials. The gray rectangle illustrates the presentation time of the go and catch cues, respectively. Hit rates and false alarm rates of crow C (B), crow E (D), and crow Y (F) and corresponding *d′*-values (log-linear adjusted) are presented. The dashed line denotes *d′* = 1.8 (C, E, G). (H) Histogram of vocalization response times. (I) Vocalization response times in hit trials plotted in relation to the waiting period duration. Means ± SEM (shaded) are depicted (100 ms bins). Underlying data can be found in S1 and S1a at https://doi.org/10.6084/m9.figshare.7571795.

For each of the three crows, the data from 10 sessions over 10 consecutive days were analyzed. All three crows vocalized consistently in response to the go cue across all 10 sessions. **Fig 2A** shows the detailed performance of crow C in a randomly chosen testing session with 300 vocalizations. The subject was reliable and temporally precise in its response to the variable go cue onset; in this example session, crow C’s hit rate was 92.0%. Failure to vocalize in a go trial was rare (“miss”). No erroneous vocalizations were produced during any of the catch trials.

This pattern of behavior was representative of the general performance of all three crows (**Fig 2B–2G**). With a mean number of vocalizations per session of 282.0 ± 29.0 (SEM) for crow C, 142.4 ± 33.7 for crow E, and 129.1 ± 27.6 for crow Y, the crows produced mean hit rates per session of 90.8% ± 0.5% (SEM) (crow C), 94.0% ± 0.3% (crow E), and 91.4% ± 0.6% (crow Y). The average miss rates were 8.5% ± 4.6% for crow C, 4.2% ± 2.4% for crow E, and 5.6% ± 2.9% for crow Y. Notably, not one of the three crows vocalized during a catch trial across any of the 10 sessions, resulting in a false alarm rate of 0% for all crows and sessions (**Fig 2B, 2D and 2F**). As a consequence, the sensitivity measure *d′*, derived from signal detection theory [27], was significantly above the threshold value of 1.8 (Fisher–Pitman exact permutation test, *p*s < 0.001) for all three crows (**Fig 2C, 2E and 2G**).

### Reaction times show no signs of response timing by crows

The crows showed a median vocalization response time of 1,688 ms (crow C), 1,957 ms (crow E), and 1,747 ms (crow Y) (**Fig 2H**). Crow C exhibited a bimodal reaction time distribution because it sometimes produced a low-intensity vocalization below the threshold of the vocal detector, followed by a second vocalization when the first vocalization yielded no reward.

Although the variable waiting period was implemented to prevent the crows from being able to respond after a fixed time interval had elapsed, we additionally checked whether the crows might have timed their vocalizations in relation to trial onset. For a timing strategy, such as “vocalize 4 seconds after the trial starts,” one would expect the vocalization response times to the presentation of the go cue to be shorter after longer waiting periods than after shorter ones. Response times were modelled to predict reaction time based on duration of the waiting period. A repeated-measures regression (with random individual crow slope and intercept) revealed that reaction time was not associated with duration of the waiting period: *F*(1,5231) = 10.56, *p* = 0.091, marginal *R*² = 0.013 (**Fig 2I**).

### High performance in a second go/nogo task with reversed colors

**Fig 3:**
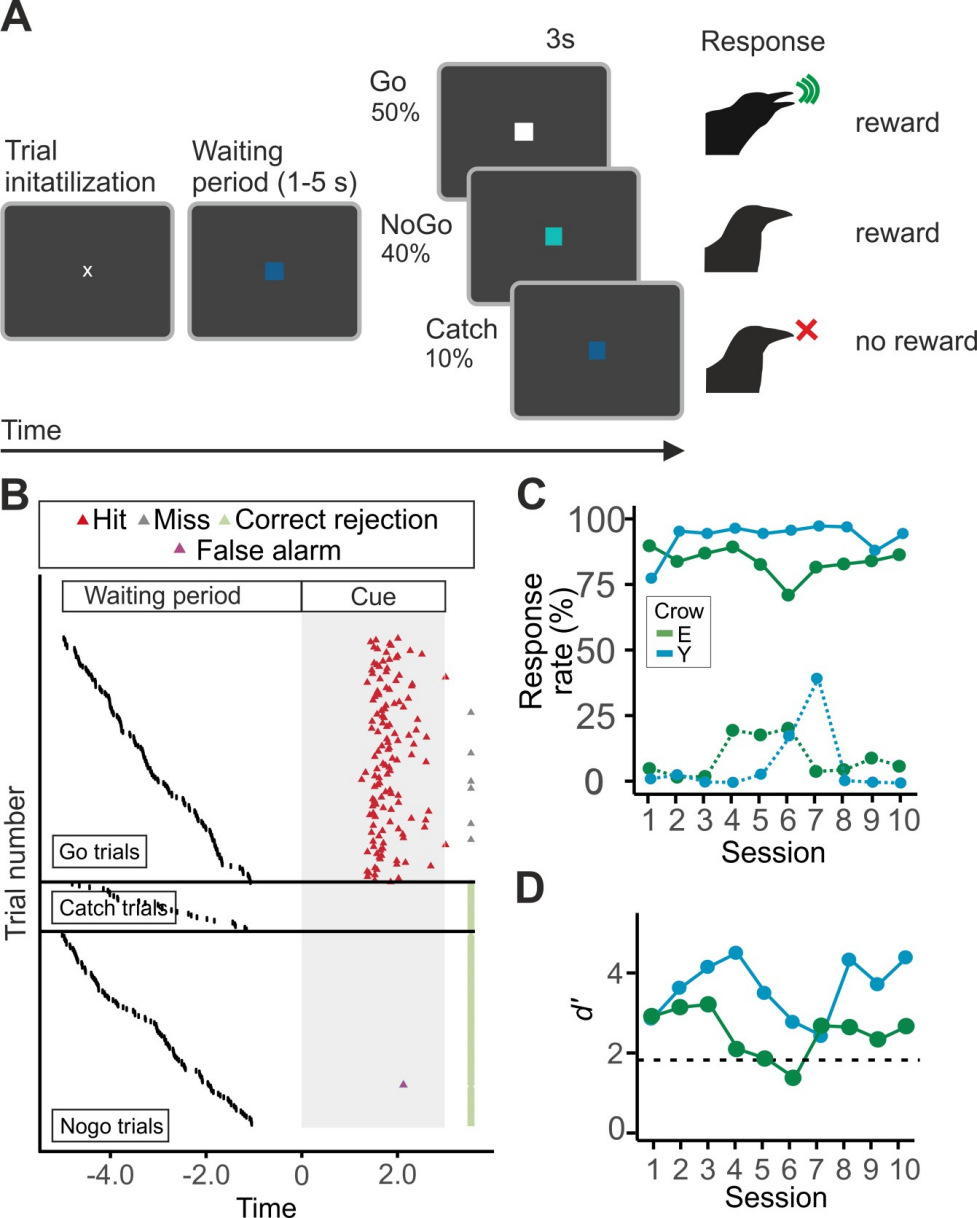
Performance in the second go/nogo task. (A) Schematic illustration of the behavioral protocol of the second task. The procedure was the same as in the first task, except that the colors of the cues were the reverse: the go cue was now white, and catch trials were blue. The novel nogo cue was turquoise (RGB values: 64, 224, 208). (B) Detailed behavior of crow Y in an exemplary session (session 2). The responses for all trials, sorted according to length of the waiting period (black dots), are shown. Each line represents one trial. Data are separated into go trials (top), catch trials (middle), and nogo trials (bottom). The small gray rectangles illustrate the presentation times of the nogo, go, and catch cues, respectively. Hit rates and false alarm rates (C) of crow E (blue) and crow Y (green) and corresponding *d′*-values (D; log-linear adjusted). The dashed line denotes *d′* = 1.8. Underlying data can be found in S2 at https://doi.org/10.6084/m9.figshare.7571795. RGB, red–green–blue.

As an additional test, we next presented two of our crows with a second task, in which the colors of the cues were reversed such that the new go cue was now the color of the previous catch cue, and vice versa. Additionally, we introduced nogo trials in which the birds were rewarded for refraining from vocalizing (see **Fig 3A** for this new protocol). The cue presented during the waiting period was blue, and the go cue was white. The nogo cue was turquoise. In nogo trials (40% of trials), the birds had to wait and refrain from vocalizing for 3 seconds, and they were rewarded for correct rejections. In total, 50% of trials were go trials, and 10% were catch trials. This new protocol was conducted to ensure that (1) any response to the blue square was not only due to a special saliency associated with blue and that (2) the vocalization was not due to an arousal elicited by the anticipation of the reward in a go trial.

Both crow E and Y learned the new task quickly. It took them 3 days (548 and 626 trials, respectively) to learn that the previous go cue was now indicating the waiting period, and vice versa. Crow Y learned the go/nogo task in another 4 days (631 trials); crow E took 12 days (2,442 trials). After retraining, both crows participated in 10 test sessions. Both crows showed high performance, with *d′* significantly above the threshold value of 1.8 (Fisher–Pitman exact permutation test; *p* = 0.002 and *p* = 0.010 for crow E and Y, respectively). As can be seen in the randomly chosen example session of crow Y (**Fig 3B**), vocalizations were limited to go trials, with only a handful of instances in nogo trials (5 false alarms in 193 trials). Because of the shorter training and increased complexity of this task, both crows’ performance was more variable than in the first task.

### Comparing cued and task-unrelated vocalizations

**Fig 4:**
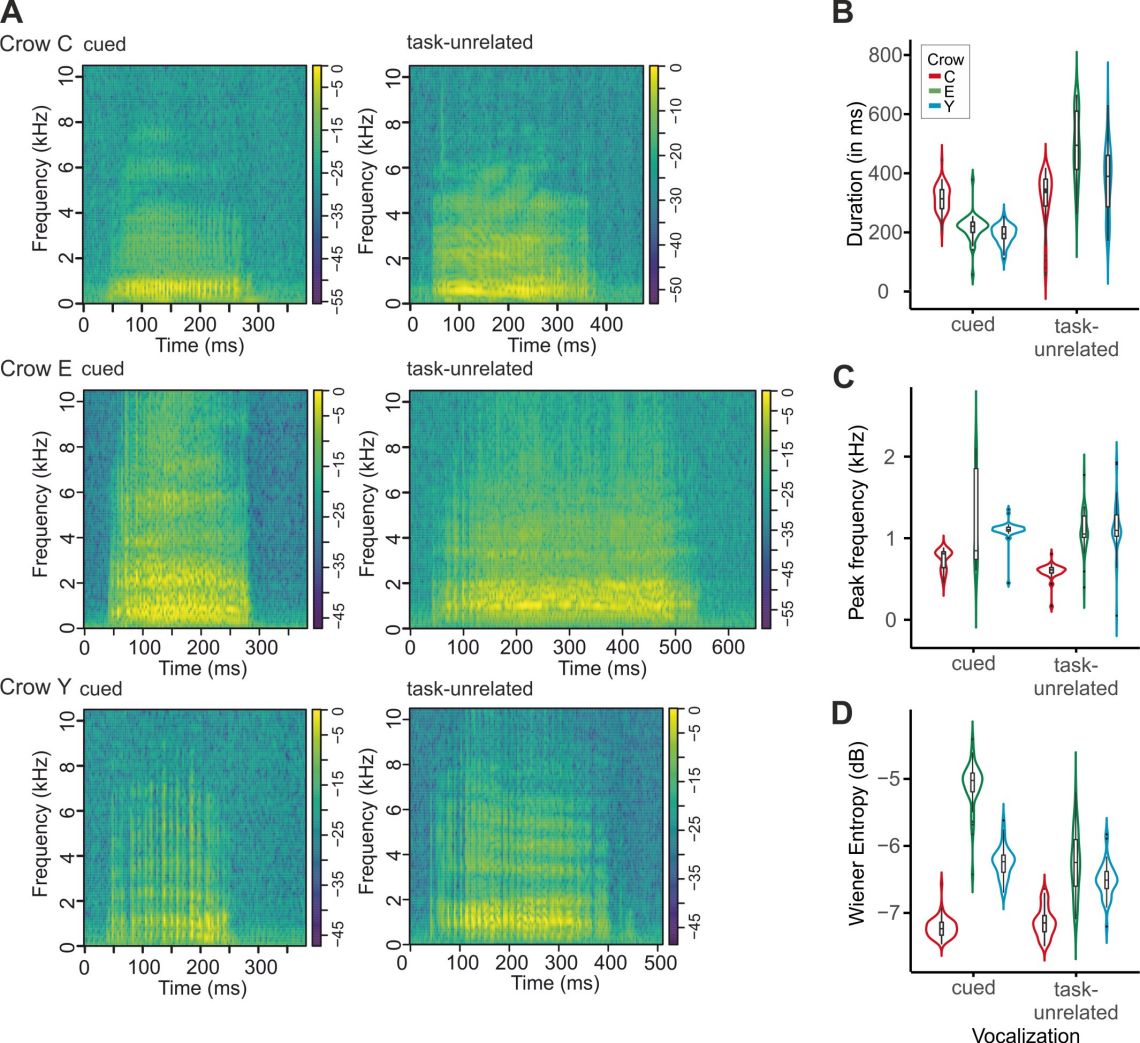
Exemplary cued and task-unrelated vocalizations as well as vocalization features. Spectrograms (A) of exemplary cued and task-unrelated vocalizations of crow C (upper row), crow E (middle row), and crow Y (lower row). (B) Box (lower and upper hinges correspond to 25th and 75th quartile, the bold black line to the median) and violin plots showing the probability density of the duration of volitional vocalizations for cued and task-unrelated vocalizations for all three birds. (C) Mean peak frequency ± SEM (shaded) of cued and task-unrelated vocalizations for all three crows. (D) Box and violin plots of entropy (in dB) of cued and task-unrelated vocalizations for all three crows. Underlying data can be found in S3, and audio files containing the example vocalizations in A1–A3 are at https://doi.org/10.6084/m9.figshare.7571795.

The volitional vocalizations of the three crows during hit trials showed some variance, both within and across crows. **Fig 4A** shows exemplary spectrograms of one randomly chosen cued vocalization of each crow (audio S1 to S3). Crow C’s vocalizations were, on average, 317 ± 52 ms in duration, crow E’s vocalizations were 214 ± 38 ms in duration, and crow Y’s vocalizations were 190 ± 26 ms in duration. The distribution of vocalization duration differed significantly between the crows (two-sample Kolmogorov–Smirnov test, *n*s = 100, *d*s = 0.47–0.92, alpha of 0.05 Bonferroni corrected for three comparisons, *p*s < 0.001). Similarly, the mean vocalization peak frequency differed significantly between the crows (two-sample Kolmogorov–Smirnov test, *n*s = 100, *d*s = 0.43–0.98, alpha of 0.05 Bonferroni corrected for three comparisons, *p*s < .001), and so did the average vocalization entropy (two-sample Kolmogorov–Smirnov test, alpha of 0.05 Bonferroni corrected for three comparisons, *n*s = 100, *d*s = 0.91–0.99, *p*s < 0.001).

We next compared cued and task-unrelated vocalizations that the crows emitted outside the task context—that is, in between trial breaks. One exemplary task-unrelated vocalization for each crow is given in **Fig 4A** (see audio S4 to S6). Task-unrelated vocalizations showed in general more variation and were longer than the cued vocalizations (see **Fig 4B and 4C**): For crow C, there was a difference in duration, peak frequency, and entropy (*n* = 61, *d* = 0.29, *p* = 0.011; *d* = 0.63, *p* < 0.001; and *d* = 0.27, *p* = 0.018, respectively). For crow E, there was a difference in duration, entropy, and peak frequency (*n* = 43, *d* = 0.97, *p* < 0.001; *d* = 0.47, *p* < 0.001; and *d* = 0.86, *p* < 0.001, respectively). For crow Y, there was a difference in duration and entropy (*n* = 42, *d* = 0.79, *p* < 0.001 and *d* = 0.57, *p* < 0.001, respectively) but none in peak frequency (*n* = 42, *d* = 0.26, *p* = 0.11). Thus, the vocalizations of all three crows emitted outside the task context differ from the vocalizations they use to respond to the cue in the task.

## Discussion

Our results demonstrate that carrion crows can volitionally control vocal output in a goal-directed manner. The vocal behavior of the crows match all three criteria required for “volitional vocalizations” outlined in the Introduction: First, the crows vocalized reliably in response to flexible visual cues (colored squares) that had no inherent meaning. Second, the crows gave temporally precise responses after the instruction cue was presented. Third, the crows withheld vocalizations in the absence of a vocalization-cuing stimulus, and they also withheld vocal output in the presence of a different cue that prohibited a vocalization.

We also show that the vocalizations are not elicited by a specific kind of cue only, but that the crows can quickly learn to vocalize to a different cue. The crows managed this fast reassociation even though in this new task the cues changed their meaning from withholding to eliciting a vocalization, and vice versa. This finding lends support to the notion that our crows’ vocal control is highly flexible. Additionally, we could exclude the possibility that the crows’ vocalizations were the result of an arousal produced by an anticipation of a food reward associated with a correct response because they did not vocalize in nogo trials even though the nogo cue was associated with a food reward. Finally, we observed that the acoustic characteristics of the vocalizations (in terms of call peak frequency, entropy, and duration) of all three crows emitted during the task differed compared with those elicited outside of the task context. This finding suggests that crows do not use stereotyped vocalizations during the cued task but rather learn to adjust the acoustic output as needed.

Our results build on previous work demonstrating vocal flexibility in songbirds. As previously mentioned, songbirds are flexible in the timing and structure of their vocalization [9,10], and reinforcement will alter performance of vocalizations even in the fully developed song of adult birds [28]. Additionally, vocalizations seem to change as a function of context, such as the size of a predator [12]. A range of previous reports in corvids specifically suggested vocal control. Ravens, for example, adjust their alarm calls depending on the composition of an audience during confrontations with dominant conspecifics such that they reduce their call rate when bonding partners of their attacker are present [29], suggesting that vocalizations are important in managing conflicts [30]. They also emit specific calls to attract conspecifics to a feeding site, which may indicate that they can use vocalizations to refer to specific situations [14]. This variability in avian vocalization can, however, be explained by an involuntary response to salient events, such as the answering of a conspecific’s call or the arousal induced by the sight of a predator or food.

Previous elegant studies have shown that birds and other nonprimate species can be conditioned to modify vocal output, albeit based on learning mechanisms that do not necessarily pertain to volitional mechanisms. For example, zebra finches can be trained to respond to a conspecific’s call (a highly affective stimulus) [31] or to shift the pitch of their vocalizations in an adaptive fashion to avoid disruption [28]. Moreover, an African grey parrot learned to utter human speech sounds to denote objects and categories [32], budgerigars were conditioned to modify their sounds to match a template [33], and bats were trained to elicit social calls in a new context to receive a reward [34]. Despite the undisputed significance of these studies in the realm of vocal-production learning, they do not address the question of volitional vocal control and do not fulfill the list of criteria outlined in the Introduction.

Our results in the carrion crow significantly extend this line of research. We explicitly show that corvid songbirds can exert volitional control over their vocal output on command. Crucially, the crows’ vocalization was initiated in the absence of any affective cues in our study and, hence, was decoupled from the accompanying motivation states of, for example, the sight of food or aggressors. Beyond the domain of vocalizations, decoupling from motivational states has previously been found in the context of caching in California scrub jays [35] and courtship food sharing in Eurasian jays [36].

The only other species that has been shown to master volitional controls in the same controlled go/nogo task is the rhesus macaque of the Primate order [37,38,39,40]. Similarly to the monkeys, our crows were able to instrumentalize and precisely time vocal utterances to receive a food reward. This result is interesting from an evolutionary point of view because, in contrast to primates that possess a layered cerebral cortex as the highest cognitive control structure, birds have an independently evolved endbrain design with a more nuclear circuit organization [41,42]. Volitional control of vocalizations therefore seems to have evolved at least twice during evolution, constituting a fascinating case of convergent evolution.

The results presented here have another interesting implication because of the close relatedness of songs and calls. Notably, songs and calls rely on some of the same mechanisms, as call plasticity in zebra finches seems to be related to the forebrain song nuclei [9,43,44,45,46,47]. Recent research has also shown that begging calls are developmental precursors for learned vocalizations [48]. In the past, the song system of songbirds has largely been studied as a self-contained neuronal machinery composed of dedicated song nuclei for the perception, learning, and production of vocalizations [2]. One of the song nuclei responsible for (among other things) structuring vocal output and ultimately controlling the songbird syrinx is the HVC (acronym used as a proper name) at the apex of the song motor system [9,49]. Currently, it is not known whether the HVC receives projections from higher-association brain areas. The present study, however, raises the question of whether vocalizations of songbirds might also be controlled by endogenous top-down influences from more cognitive areas. The pallial endbrain area, called “nidopallium caudolaterale” (NCL), would be a suitable candidate for providing a source of executive vocal control. The NCL is a high-level cognitive endbrain structure in birds and is considered to be the functional equivalent of the mammalian prefrontal cortex [50]. Neurons in the crow NCL are involved in rule switching [51], abstract categorization [52], and cross-modal associations [53]. The present study opens then the question of whether the NCL is also involved in the control of vocalizations. Relatively little is known, however, about whether and how the NCL is connected to the song system. Interestingly, the HVC has been suggested to be a songbird specialization of the NCL [54,55]. Both NCL and HVC control and initiate learned sequences, either of motor sequences in pigeons (NCL [55]) or song elements in zebra finches (HVC [56]). Topographic projections also seem to connect the NCL with the robust nucleus of the arcopallium (RA) [57]. Hence, it seems plausible that the NCL itself is connected to song nuclei HVC and RA and might be involved in the control of vocalizations in oscine birds. Consequently, further work is needed to evaluate a putative neurobiological basis of avian cognitive vocal control.

A new line of research capitalizing on the brain’s neuromodulatory systems also demonstrates that the song system is affected by networks outside the classical song system. Recent findings in zebra finches show that nuclei of the song system are under the influence of dopamine, which not only signals performance errors in singing birds [58] but also helps to encode the cultural transmission of vocal behavior [59]. Although dopamine is well known to play a role in reward-based learning, research in primates shows that it also impacts cognitive control functions in the prefrontal cortex [60]. It therefore stands to reason that dopamine also plays a more general role in corvid cognitive control and, specifically, the volitional production of vocalizations.

## Materials and methods

Data were collected from three male carrion crows (*C*. *corone corone*), aged 8–10 months during data collection using the first task (crow C, E, and Y) and aged 20–22 months during retraining and data collection for the second task (only crow E and Y were used for this second experiment, as crow C became engaged in a long-lasting electrophysiological recording project and therefore was no longer available). The experiments were approved by the local authorities in charge (Regierungspräsidium Tübingen and Landratsamt Tübingen, license ZP 3/15), conducted in accordance with German and European law and the Guidelines for the Care and Use of Laboratory Animals of the National Institutes of Health, and carefully monitored by the veterinary service of University of Tübingen. The crows were housed in large indoor aviaries (360 × 240 × 300 cm) side by side in a group of three at the Animal Physiology Unit, University of Tübingen. The crows had been taken from the institute’s breeding stock in May 2017 and were hand raised. The crows were kept on a controlled feeding protocol for the duration of the experiment and earned food during the daily tests. If necessary, food was supplemented after the tests. Body weight was measured daily. Water was provided ad libitum in the aviary and during testing.

### Apparatus

The crows were trained and tested in a darkened operant conditioning chamber [61]. The chamber was coated with sound-attenuating foam mats. Stimuli were presented on a touch screen monitor (3 M Microtouch, 15”, 60 Hz refresh rate). The CORTEX program (provided as freeware available at ftp://ftp.cnl.salk.edu/pub/cortex/, National Institute of Mental Health, Bethesda, MD, USA) was used for stimulus presentation and measuring the crows’ performance. Vocalizations were classified online using a custom-built MATLAB program and recorded using a Sennheiser MKE 600 microphone with a sampling rate of 40,000 Hz for offline analysis. Rewards (bird food pallets or larvae of the mealworm beetle) for correct trials were delivered with an automated feeder below the screen. Additionally, crows received auditory feedback for correct responses. Leather jesses secured crows loosely to a perch placed in front of the monitor. An infrared light barrier, in combination with a reflector foil attached to the crows’ head, registered when the crow was positioned in front of and facing the screen. The retainer of the reflector of the light barrier was implanted under general anesthesia (for a description of surgical procedures, see [62]).

### Behavioral protocol

Crows were trained on a detection task in which they had to vocalize in response to the detection of a visual go cue to receive a reward. We started rewarding the crows at the age of 3 months by exploiting social contact vocalizations during human–crow interactions. First, vocalizations in all contexts were rewarded by the experimenter with food. After vocalizations were emitted reliably, we transferred the behavior to the operant chamber they were tested in. Here, we started rewarding crows automatically using a feeder and only when a blue square, the go cue, was presented on the screen. At the beginning of this regime, the crows were prompted to emit a vocalization—for example, by calling their name or by showing them the experimenter’s face and hand.

Once crows reliably vocalized during the presentation of the cue, we introduced the waiting cue preceding the go cue. Vocalizations during the waiting cue were “punished” with a short time-out, and hits were rewarded. Correct rejections as well as misses were neither rewarded nor punished. Once crows had a stable hit rate of over 80%, the catch trials and variability in the length of the waiting period were introduced. In a last step, the crows had to use the infrared light barrier to start a trial. The crows had to stay in this light barrier during the waiting cue and the first 300 ms after go cue onset; afterward, the crows were free to move the head during vocalizing. Trials were aborted when the crow left the light barrier too early—i.e., during the waiting cue. Time-outs were indicated by a 100 ms flash of the screen; then, the screen stayed dark, and the possibility to start a new trial was delayed. The final task procedure is depicted in **Fig 1**, with a waiting period of 1–5 seconds (randomized) followed by the go cue for 3 seconds.

In a second experiment, we presented crows with a modified task (task procedure depicted in **Fig 3B**) in which the colors of the cues were reversed: the color of the new go cue was now the color of the previous catch cue, and vice versa. In addition, nogo trials were introduced in which the birds were rewarded for refraining from vocalizing.

### Analysis

Performance and reaction times were collected online and analyzed offline using MATLAB and R. Vocalizations during go trials were defined as “hits,” and vocalizations during catch trials were defined as “false alarms” in the detection paradigm. Sensitivity values *d*′ derived from signal detection theory [27] were calculated by subtracting *z*-scores (normal deviates) of median “hit” rates from *z*-scores of median “false alarm” rates (*d*′ = *z*[hit rate] − *z*[false alarm rate]). Because the selectivity measure *d′* relies on both correct responses (“hits”) and “false alarms,” putatively variable spontaneous-call emission rates were taken into account. Because of false alarm rates of 0% in all crows, *d*′-estimates were corrected by a log-linear approach in which 0.5 is added to the frequency of false alarms in each cell of the contingency table [63]. Whether *d′* was above the threshold value of 1.8 was calculated with a Fisher–Pitman permutation test [64] (R package “coin”) for each crow separately. The regression to examine the relationship between waiting times and reaction times was calculated with the R package “lme4,” and *R*² marginal was calculated using the R package “MuMIn” [65].

Peak frequency and Wiener entropy were calculated using the R packages “seewave” [66] and “soundgen” [67]. Because the assumptions of normality and homogeneity of variance were not met, two-sample, two-tailed Kolmogorov–Smirnov tests were used to compare distributions of the durations, mean peak frequencies, and Wiener entropy between crows. Specifically, the Kolmogorov–Smirnov test allows for a nonparametric comparison of the shape of a distribution [68]. For analysis, we used the same number of vocalizations for each bird (*n* = 100). Features of the vocalizations were also compared between cue-elicited vocalizations and task-unrelated vocalizations with two-tailed Kolmogorov–Smirnov tests. To this end, vocalizations were collected in two to four consecutive sessions after the initial data collection period had ended; hence, we only compared cued vocalizations that were collected in the same period in which the task-unrelated ones were collected (April 2019). For analysis, we matched the number of cued vocalizations to the number of task-unrelated vocalizations for each crow (*n* = 61, *n* = 43, and *n* = 42, respectively). In order to compare the vocalizations of different individuals with each other, we treated each vocalization as an independent observation, in line with analyses in previous studies [37,40].

## Abbreviations

NCL: nidopallium caudolaterale
PC: personal computer
RA: robust nucleus of the arcopallium
RGB: red–green–blue
